# A comparison of the endotoxin biosynthesis and protein oxidation pathways in the biogenesis of the outer membrane of *Escherichia coli* and *Neisseria meningitidis*

**DOI:** 10.3389/fcimb.2012.00162

**Published:** 2012-12-20

**Authors:** Susannah Piek, Charlene M. Kahler

**Affiliations:** Department of Pathology and Laboratory Medicine, The University of Western AustraliaPerth, WA, Australia

**Keywords:** oxidoreductases, disulfide bonds, protein oxidation, protein isomerization, lipopolysaccharides (LPS), lipooligsaccharides (LOS), *Neisseria meningitidis*

## Abstract

The Gram-negative bacterial cell envelope consists of an inner membrane (IM) that surrounds the cytoplasm and an asymmetrical outer-membrane (OM) that forms a protective barrier to the external environment. The OM consists of lipopolysaccahride (LPS), phospholipids, outer membrane proteins (OMPs), and lipoproteins. Oxidative protein folding mediated by periplasmic oxidoreductases is required for the biogenesis of the protein components, mainly constituents of virulence determinants such as pili, flagella, and toxins, of the Gram-negative OM. Recently, periplasmic oxidoreductases have been implicated in LPS biogenesis of *Escherichia coli* and *Neisseria meningitidis*. Differences in OM biogenesis, in particular the transport pathways for endotoxin to the OM, the composition and role of the protein oxidation, and isomerization pathways and the regulatory networks that control them have been found in these two Gram-negative species suggesting that although form and function of the OM is conserved, the pathways required for the biosynthesis of the OM and the regulatory circuits that control them have evolved to suit the lifestyle of each organism.

## The Gram-negative cell envelope

The Gram-negative bacterial cell envelope consists of an inner membrane (IM) that surrounds the cytoplasm and an asymmetrical outer-membrane (OM) that forms a protective barrier to the external environment (reviewed in Ruiz et al., 2006). The two membranes are separated by an aqueous compartment or periplasm which contains a thin peptidoglycan layer that contributes to cell structure and resistance to osmotic stress. Unlike the cytoplasmic compartment, the periplasm is an oxidizing environment that is devoid of ATP and comprises 10% of the cell volume. The Gram-negative OM coordinates interactions with the external environment, preventing the entry of toxic molecules while still allowing intake of nutrients and excretion of toxic waste products. The asymmetric OM consists of an inner leaflet composed of phospholipids and an outer leaflet composed of lipopolysaccahride (LPS), which is responsible for the highly efficient barrier function of the OM (Raetz and Whitfield, 2002). There are two types of proteins incorporated in the OM; outer membrane proteins (OMPs) and lipoproteins, which usually face the periplasm and form complexes with OMPs and periplasmic machinery. OMPs known as porins selectively allow intake of nutrients and expel toxic waste. OMPs can also have enzymatic functions and act as adhesins. As a result, the LPS and protein components of the OM have important roles in the survival and virulence of Gram-negative bacteria.

Oxidative protein folding mediated by periplasmic oxidoreductases is required for the biogenesis of the protein components, mainly constituents of virulence determinants such as pili, flagella, secretion systems and toxins, of the Gram-negative OM (Kadokura and Beckwith, 2010). Oxidoreductases, known as Disulfide bond (Dsb) proteins, catalyze disulfide bond formation in membrane and secreted proteins as they transit through the periplasm to the OM. Disulfide bonds are covalent linkages formed between thiol groups of two Cys residues and are necessary for the stability and/or activity of proteins. Recently, periplasmic oxidoreductases have been implicated in LPS biogenesis of *Escherichia coli* (Denoncin et al., 2010) and *Neisseira meningitidis* (Piek et al., 2012), suggesting a much larger role of these enzymes in OM biogenesis.

The structure and composition of the OM is conserved in all Gram-negative bacteria, regardless of the ecological niche of the organism. Comparative genomic studies have shown that epidemic pathogenic bacteria have reduced genomes both in size and regulatory complexity when compared to organisms that have a commensal lifestyle (Merhej et al., 2009; Georgiades, 2012). Pathogenic bacteria also acquire many virulence determinants which enable specialized niche adaptation. A recent study by Liechti and Goldberg (2012) has shown that genome reduction and niche specialization in *H. pylori* has resulted in a smaller number of components required for OMP, LPS, and lipoprotein transport pathways. In this review, the periplasmic protein folding and LPS biogenesis pathways of the extensively studied commensal *E. coli*, and epidemic pathogen, *Neisseria meningitidis*, are compared. Differences in OM biogenesis, in particular the transport pathways for endotoxin to the OM, the composition and role of the protein oxidation and isomerization pathways for protein folding in the periplasm and the regulatory networks that control them are elucidated. It appears that although form and function of the OM is conserved, the pathways required for the biosynthesis of the OM and the regulatory circuits that control them have evolved to suit the lifestyle of each organism.

## The Gram-negative cell envelope of *E. coli*

### Periplasmic oxidoreductases and their role in OM biogenesis in *E. coli*

**Figure 1:**
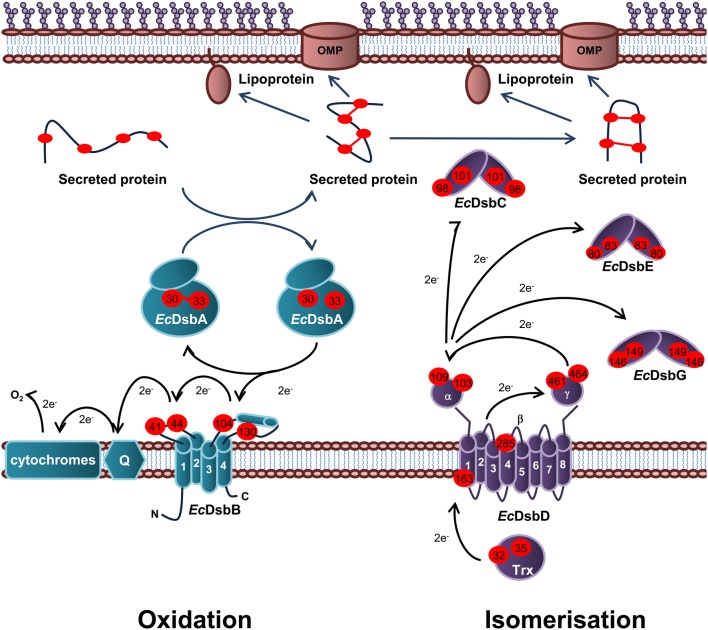
**Oxidation and isomerization pathways of periplasmic protein folding in *E. coli*.** Oxidation pathway: The soluble oxidoreductase *Ec*DsbA catalyses disulfide bond formation between adjacent cysteine residues of secreted proteins. *Ec*DsbA is re-oxidized by the IM bound *Ec*DsbB. Under aerobic growth conditions, electrons flow from *Ec*DsbB to molecular oxygen via ubiquinone (Q) and cytochrome. Isomerization pathway: The oxidoreductase *Ec*DsbC re-shuffles any incorrect disulfide bonds which is important in secreted proteins that contain multiple disulphide bonds between non-consecutive cysteine residues. *Ec*DsbC, *Ec*DsbG, and *Ec*DsbE are kept in a reduced state by the IM bound *Ec*DsbD which in turn is reduced by passing electrons to thioredoxin in the cytoplasm. The oxidation pathway is in turquoise and the isomerization pathway is in purple. Cysteine residues are denoted by a red circle containing the residue number. The direction of electron transfer is shown by black arrows while protein folding is shown with dark blue arrows.

Periplasmic protein folding catalyzed by Dsb proteins is best characterized in *E. coli* K12. Dsb proteins ensure correct disulfide bond formation through two independent but parallel pathways of periplasmic protein folding known as the oxidation and isomerization pathways. The oxidation pathway (DsbA/DsbB system) is primarily responsible for the catalysis of disulfide bond formation between adjacent Cys residues of protein substrates while the isomerization pathway (DsbD/DsbC system) re-shuffles any incorrectly formed disulfide bonds, which is important if the native protein contains multiple disulfide bonds between non-consecutive Cys residues (Figure 1).

#### Oxidation pathway of periplasmic protein folding in E. coli

The oxidation pathway is required for the correct folding of a number of virulence proteins of *E. coli*. In fact, there are approximately 300 disulfide-containing proteins that transit through the periplasm of *E. coli* (Hiniker and Bardwell, 2004; Dutton et al., 2008) and are therefore substrates of the oxidation pathway of periplasmic protein folding. Central to this pathway is the oxidoreductase *Ec*DsbA; the primary disulfide donor that interacts with these substrates (Bardwell et al., 1991). *Ec*DsbA is kept in an oxidized and active state by the membrane bound *Ec*DsbB (Bardwell et al., 1993), which in turn is re-oxidized by passing electrons via quinones (Q) to the electron transport pathway in the cytoplasmic membrane (Bader et al., 1999; Kobayashi and Ito, 1999). There are a number of features of both *Ec*DsbA and *Ec*DsbB that drive the flow of electrons from the reduced substrate through to the electron transport pathway.

*Ec*DsbA is a monomeric 21 kDa periplasmic enzyme that belongs to the thioredoxin superfamily (Martin et al., 1993a). Features common to this superfamily include an active site motif consisting of two Cys residues separated by two amino acids (CXXC) embedded in a thioredoxin-like fold (Martin, 1995). *Ec*DsbA differs from other members of the thioredoxin superfamily in that it contains an extra alpha (α)-domain (65 residues forming four α-helices) inserted into the center of the thioredoxin domain. The α-domain forms a globular-like cap over the active site (C_30_PHC_33_) located at the N-terminus of the first α-helix of the thioredoxin domain (Martin et al., 1993a). Oxidized *Ec*DsbA contains a disulfide bond between the two Cys residues of the active site which is donated to an unfolded protein substrate during protein oxidation. *Ec*DsbA-substrate disulfide exchange proceeds as a biomolecular nucleophilic substitution reaction.

*Ec*DsbA is an extremely efficient oxidizing disulfide catalyst that rapidly oxidizes substrate proteins. With a standard redox potential of ~−120 mV, *Ec*DsbA is one of the strongest thiol oxidants (Huber-Wunderlich and Glockshuber, 1998), which can be explained in part by biophysical properties. The active site C_30_ is surface exposed and has an unusually low p*K*_a_ of ~3.3 (the normal p*K*_a_ of Cys is ~9) (Nelson and Creighton, 1994; Huber-Wunderlich and Glockshuber, 1998). As C_30_ is a thiolate anion at physiological pH, the oxidized form of *Ec*DsbA is less stable than the reduced form and is therefore more reactive. This drives the thermodynamic flow of electrons from the substrate to *Ec*DsbA and the transfer of the disulfide from the *Ec*DsbA active site to the substrate.

Another important structural feature of *Ec*DsbA is the loop between α6 and β4, which contains the highly conserved *cis*-Pro_151_ residue that impacts directly on the active site motif and forms the other half of the active site in most members of the thioredoxin superfamily (Martin et al., 1993b; Martin, 1995). Mutating the *cis*-Pro_151_ to a Thr resulted in accumulation of mixed-disulfide intermediate complexes, suggesting a role of *cis*-Pro_151_ in *Ec*DsbA substrate release (Kadokura et al., 2004). The residue immediately preceding *cis*-Pro_151_ in *Ec*DsbA (V_150_) modulates redox potential and coordinates interactions with *Ec*DsbB and substrate proteins. In addition, the solved structure of *Ec*DsbA in a complex with a peptide substrate revealed that the residues of the loop between α6 and β4, V_145_QLRGV_150_, as well as the residues of the type IV β turn between β3 and α2, F_63_MGG_66_, form the substrate binding surface of *Ec*DsbA (Paxman et al., 2009). Interestingly, these residues have also been implicated in interaction with *Ec*DsbB.

*Ec*DsbB (20 kDa) consists of four trans-membrane α-helices connected by two periplasmic loops that contain two intra-molecular disulfide bonds, between C_41_ and C_44_ in the first periplasmic loop, and C_104_ and C_130_ in the second periplasmic loop, that are essential to *Ec*DsbA oxidation (Jander et al., 1994; Kadokura and Beckwith, 2002). Co-crystallization of the *Ec*DsbA (C33A) in a covalently linked complex with *Ec*DsbB (C130S) revealed the residues P_100_FATCDF_106_ of the second periplasmic loop of *Ec*DsbB is accommodated in the hydrophobic groove of *Ec*DsbA (Inaba et al., 2006a). This structure also revealed that the *Ec*DsbB residues C_104_DF_106_ interact with the residues R_148_GV_150_ of *Ec*DsbA to form a short anti-parallel β-sheet, similar to the interaction between *Ec*DsbA and substrates (Paxman et al., 2009). It appears that the relative specificity of reduced *Ec*DsbA for *Ec*DsbB could be a result of the additional interactions of *Ec*DsbB with residues located within the hydrophobic groove of *Ec*DsbA. Recent NMR analysis of the *Ec*DsbA homolog in *Vibrio cholorae* (*Vc*DsbA) revealed greater interdomain flexibility resulting in the widening of the hydrophobic groove in the reduced form of the enzyme (Horne et al., 2007). This suggests that the reduced form of *Ec*DsbA could be in a more open conformation allowing *Ec*DsbB interaction with the hydrophobic groove.

The transfer of electrons from *Ec*DsbA to *Ec*DsbB is an energetically unfavorable reaction as the standard redox potential values for the *Ec*DsbB Cys pairs (−210 mV for C_41_–C_44_ and −220 mV for C_104_–C_130_) (Inaba et al., 2005) are much lower than that of *Ec*DsbA (−122 mV for C_30_–C_33_) (Wunderlich et al., 1993). To overcome this barrier, disulfide exchange is initiated by interaction of the second periplasmic loop of *Ec*DsbB with the open hydrophobic groove of reduced *Ec*DsbA. This interaction induces a conformational change in the second periplasmic loop of *Ec*DsbB so that C_104_ and C_130_ are separated spatially, with C_130_ situated closer to the C_41_–C_44_ disulfide of *Ec*DsbB. It is thought that *Ec*DsbA induced separation of the C_104_–C_130_ disulfide of *Ec*DsbB is what makes transfer of the disulfide bond from *Ec*DsbB to *Ec*DsbA an energetically favorable reaction (Kadokura and Beckwith, 2002; Tapley et al., 2007; Inaba et al., 2009). The solved *Ec*DsbA (C33A)—*Ec*DsbB (C130S) crystal structure revealed a possible role of M_64_ of *Ec*DsbA in separation of the C_104_–C_130_ disulfide as it appeared to intervene between C_104_ and the serine at position 130 (Inaba et al., 2006a). Interestingly, this residue was also implicated in *Ec*DsbA interactions with substrates (Paxman et al., 2009). Upon separation of the C_104_ and C_130_ residues of *Ec*DsbB, the thiolate anion C_30_ of reduced *Ec*DsbA attacks C_104_ of *Ec*DsbB forming a mixed disulfide bridge (Kadokura and Beckwith, 2002). This mixed disulfide is then resolved by one of two possible pathways. In the rapid pathway, oxidized *Ec*DsbA is released prior to oxidation of *Ec*DsbB and in the slow pathway, oxidized *Ec*DsbA is released simultaneously with oxidized *Ec*DsbB (Inaba and Ito, 2008). In both pathways, C_44_ of *Ec*DsbB is transiently reduced, which leads to the formation of a charge transfer complex between *Ec*DsbB and ubiquinone (UQ) (Inaba et al., 2006a; Zhou et al., 2008).

It has been proposed that under aerobic conditions, oxidation of the C_41_–C_44_ residues of *Ec*DsbB is initiated by transfer of a partial charge to the benozoquinone ring of UQ by the thiolate anion C_44_ of reduced *Ec*DsbB. This is followed by C_44_ nucleophilic attack of UQ to form a charge-transfer complex between UQ and *Ec*DsbB that is stabilized by R_48_ of *Ec*DsbB (Kadokura et al., 2000; Kobayashi et al., 2001; Inaba et al., 2006b). The existence of a charge transfer complex is supported by quantum chemical simulation by Inaba et al. (2006b), and the observation that *Ec*DsbB elicits a characteristic red shift of bound UQ during the *Ec*DsbA oxidation (Inaba et al., 2004, 2006b). The *Ec*DsbA (C33A)—*Ec*DsbB (C130S) crystal structure supports this model for sequential disulfide exchange as the six redox-active Cys residues are arranged in a straight line. This structure also shows C_44_ of *Ec*DsbB is well positioned to form a charge transfer complex with UQ (Inaba et al., 2006a).

#### Alternatives to the classical oxidation pathway of periplasmic protein folding in E. coli

The classic protein oxidation pathway represented by *Ec*DsbA and *Ec*DsbB has been shown to be responsible for introducing disulfide bonds into the vast majority of proteins in *E. coli* (Bardwell et al., 1991). However, exceptions to this classic or generalist pathway have increasingly been found and are characterized by oxidoreductases which are specialized for the recognition of a specific or a small array of substrates. Pathways which contain specialized oxidoreductases fall into two separate categories. The first category consists of specialist oxidoreductases, which are partnered with a specific redox partner. As an example, uropathogenic *E. coli* (UPEC) and *Salmonella enterica* serovar Typhimurium contain a second functional redox pair, DsbL and DsbI, which are 19% and 24% identical in sequence to *Ec*DsbA and *Ec*DsbB, respectively. DsbL specifically oxidizes the substrate arylsulfate sulfotransferase (ASST), for which there is no virulence phenotype (Grimshaw et al., 2008; Lin et al., 2009). The genes encoding DsbL, DsbI, and ASST are organized in a tricistronic operon that is found throughout *Salmonella* and in a subset of Enterobacteriacea (Grimshaw et al., 2008). Interestingly, DsbL and DsbI act as a specific redox pair that is independent and does not interact with the classic pathway (in other words, DsbL cannot ultilize *Ec*DsbB as a redox partner).

The second category of specialist oxidoreductase does not have a specialist redox partner but have a reduced substrate repertoire. In this example, *S. enterica* serovar Typhimurium contains a virulence plasmid encoding a third DsbA-like protein, *Se*SrgA (SdiA-regulated gene) (Bouwman et al., 2003), in addition to the chromosomally encoded specialist oxidoreductase, *Se*DsbL, and the generalist oxidoreductase, *Se*DsbA. *Se*SrgA has a narrow substrate range as it only efficiently oxidizes two substrates, PefA (plasmid encoded fimbrae) (Bouwman et al., 2003), and SpiA, a component of the Type III secretion system (Miki et al., 2004). Interestingly, *Se*SrgA does not have its own redox partner but is dependent on *Se*DsbB of the classic oxidation pathway (Bouwman et al., 2003). *Se*DsbL and *Se*SrgA contain the active site residues of CPFC and CPPC, respectively, corresponding to C_31_PHC_33_ in *Ec*DsbA, and have very different redox potentials of ~−95 mV and ~−154 mV, respectively, compared to ~−120 mV of *Ec*DsbA.

#### Isomerization pathway of periplasmic protein folding in E. coli

The isomerization (*Ec*DsbC/*Ec*DsbD) pathway is responsible for the re-shuffling of incorrect disulfide bonds introduced by *Ec*DsbA into the periplasmic proteins of *E. coli* (Rietsch et al., 1996). Incorrect disulfide bond formation may occur if the bonds need to form between non-consecutive Cys residues (Berkmen et al., 2005). As membrane and secreted proteins of prokaryotes rarely contain multiple disulfides (Hiniker and Bardwell, 2004), the formation of a single disulfide bridge between two adjacent Cys residues is often sufficient for correct protein folding. As a result, the oxidation pathway plays a much larger role in periplasmic protein folding of *E. coli* than the isomerization pathway (Yu and Kroll, 1999).

*Ec*DsbC is the major protein disulfide isomerase central to the isomerization pathway and is kept in the reduced “active form” by the IM membrane bound *Ec*DsbD which in turn is reduced by thioredoxin/thioredoxin reductase and NAPDH (Joly and Swartz, 1997; Rietsch et al., 1997). *Ec*DsbC is a V-shaped homodimer consisting of two 23 kDa monomers. The monomers consist of a C-terminal thioredoxin domain and an N-terminal dimerization domain connected by a linker sequence (McCarthy et al., 2000). The active site residues C_98_GYC_101_ [redox potential of −130 mV (Zapun et al., 1995)] are located in the thioredoxin domain and are positioned so as to face the inside of the V-shaped homodimeric structure (McCarthy et al., 2000). This surface is occupied primarily by hydrophobic residues which presumably aids interaction with a variety of substrates. In addition, *Ec*DsbC displays periplasmic chaperone activity that would facilitate substrate interactions (Chen et al., 1999). While dimerization of the *Ec*DsbC monomers does not appear to be necessary for isomerase activity of the enzyme, it is important for protection of the active site Cys residues from oxidation by *Ec*DsbB (Bader et al., 2001). Therefore, it has been suggested that the dimeric structure of *Ec*DsbC evolved to limit cross talk between the oxidation and isomerization pathways of periplasmic protein folding.

There are two models for *Ec*DsbC-mediated protein disulfide isomerization both of which begin with nucleophilic attack of the incorrect disulfide bond in the substrate by C_98_ of reduced *Ec*DsbC resulting in the formation of an *Ec*DsbC-substrate mixed disulfide intermediate complex. The intermediate complex is resolved by either attack of the mixed disulfide by a third Cys of the substrate resulting in correct disulfide bond formation in the substrate and oxidized *Ec*DsbC or by attack of the disulfide by C_101_ of *Ec*DsbC resulting in reduced substrate, that could then be correctly oxidized by *Ec*DsbA (Rietsch et al., 1997; Walker and Gilbert, 1997; Darby et al., 1998). Either way, *Ec*DsbC must be maintained in the reduced form to initiate disulfide bond isomerization.

In addition to *Ec*DsbC, *E. coli* contains two substrate specific periplasmic disulfide isomerases, known as *Ec*DsbG (Bessette et al., 1999) and *Ec*DsbE (Reid et al., 2001). *Ec*DsbC and *Ec*DsbG share 24% amino acid identity, exhibit a conserved tertiary structure and are both maintained in a reduced state in the periplasm by the membrane bound *Ec*DsbD (Bessette et al., 1999; Reid et al., 2001). However, the V-shaped cleft is much wider in *Ec*DsbG and more acidic when compared to that of *Ec*DsbC (Heras et al., 2004). It was recently shown that *Ec*DsbG preferentially interacts with periplasmic transpeptidases that cross link the major OM lipoprotein to peptidoglycan of *E. coli* (Depuydt et al., 2009). These enzymes contain a single Cys that is essential for enzymatic activity and which when attacked by oxygen radicals, will form a sulfenic acid adduct that inactivates the enzyme. It therefore appears that *Ec*DsbG plays a role in protecting proteins with a single Cys residue from sulfenylation in the periplasm. The large, hydrophilic binding cleft of *Ec*DsbG could result in preferential binding of this isomerase to large folded proteins with a single oxidized Cys residue which cannot be accessed by *Ec*DsbC. *Ec*DsbE (also known as CcmG) is required for cytochrome C biogenesis (Reid et al., 2001). For simplicity this review will focus on *Ec*DsbD reduction of *Ec*DsbC.

The IM membrane bound oxidoreductase *Ec*DsbD mediates trans-membrane electron transfer from cytoplasmic thioredoxin to periplasmic *Ec*DsbC therefore maintaining *Ec*DsbC in the reduced “active” form (Rietsch et al., 1997). *Ec*DsbD consists of a periplasmic N-terminal domain with an immunoglobulin-like fold (*Ec*DsbDα), a hydrophobic core (*Ec*DsbDβ) and periplasmic C-terminal domain (*Ec*DsbDγ) with a thioredoxin-like fold (Haebel et al., 2002; Rozhkova et al., 2004; Cho et al., 2007). Each domain of *Ec*DsbD contains two Cys residues; C_103_ and C_109_ in *Ec*DsbDα (redox potential ~−229 mV); C_163_ and C_285_ in *Ec*DsbDβ redox potential ~−246 mV); and C_461_ and C_464_ in *Ec*DsbDγ (redox potential ~−241 mV) (Rozhkova and Glockshuber, 2008). These Cys residues are essential for reduction of *Ec*DsbC (Katzen and Beckwith, 2000).

*Ec*DsbD mediated reduction of *Ec*DsbC occurs through a disulfide bond cascade from *Ec*DsbC to thioredoxin in the cytoplasm (Katzen and Beckwith, 2000; Collet et al., 2002). *Ec*DsbDβ consists of eight trans-membrane domains (TM1–TM8) with C_163_ and C_285_ present in the C-terminal regions of TM1 and TM4, respectively. Recent studies by Cho et al. (Cho et al., 2007; Cho and Beckwith, 2009) suggest *Ec*DsbDβ exhibits an hourglass-like structure with inverted symmetry in which the surfaces displaying C_163_ and C_285_ face the cytoplasm and periplasm, respectively. This arrangement appears to allow C_163_–C_285_ interaction with thioredoxin in the cytoplasm and *Ec*DsbDγ in the periplasm and therefore facilitate trans-membrane transfer of electrons (Katzen and Beckwith, 2000; Collet et al., 2002; Haebel et al., 2002; Rozhkova et al., 2004). In addition, examination of the redox potentials of each of the Cys pairs involved in reduction of *Ec*DsbC reveals transfer of electrons from thioredoxin to *Ec*DsbC to be a thermodynamically favorable reaction (Rozhkova and Glockshuber, 2008).

#### The role of periplasmic oxidoreductases in virulence of E. coli

**Figure 2:**
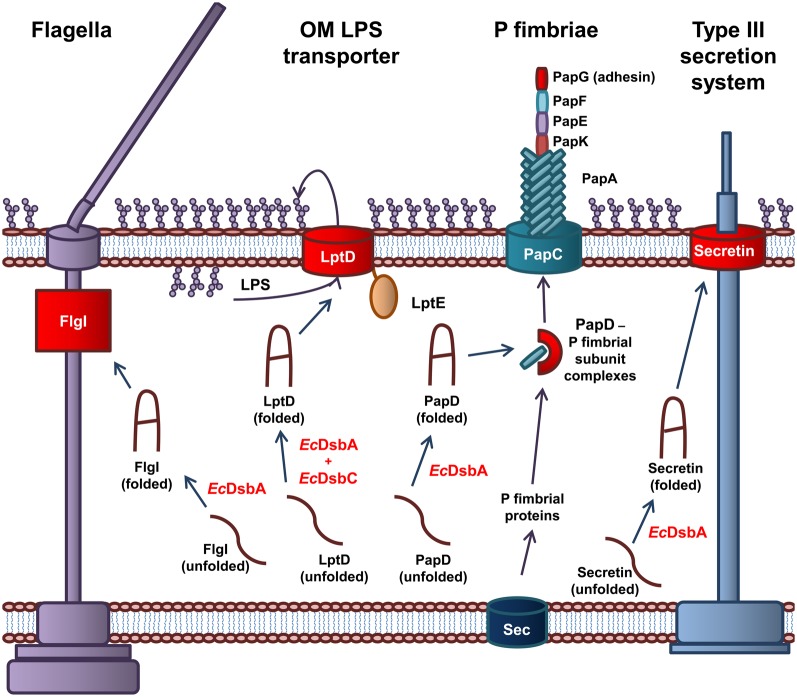
**The role of *Ec*DsbA and *Ec*DsbC in biosynthesis of virulence factors in *E. coli*.** EcDsbA catalyzes the formation of a disulfide bond in the FlgI P-ring motor protein required for assembly of functional flagella. Both *Ec*DsbA and *Ec*DsbC are required for the correct folding of the OMP LptD, which associates with the OM lipoprotein, LptE, to form the OM LPS transporter. *Ec*DsbA catalyzes the formation of a disulfide bond in the PapD chaperone, which transports the P fimbriae subunits from the Sec translocon in the IM to the usher protein PapC in the OM, which coordinates the assembly of the P fimbraie. *Ec*DsbA catalyses the formation of a disulfide bond in the OM secretin of the Type III secretion system. Protein folding reactions are shown by dark blue arrows, while cellular processes are shown by dark purple arrows.

Periplasmic oxidoreductases are required for the biogenesis of the protein component of the OM of Gram-negative bacteria. As such, they are responsible for the correct folding of virulence proteins that are expressed on or around the OM (Figure 2). *Ec*DsbA is central to the virulence of pathogenic *E. coli* as it is required for the biogenesis of machinery involved in adhesion to host cells, cellular spread, and secretion of effector molecules (Heras et al., 2009). Recently, the DsbA proteins of *E. coli* have been implicated in the biogenesis of the LPS component of the OM (Denoncin et al., 2010).

*Ec*DsbA is required for the biogenesis of virulence factors involved in adhesion and movement of pathogenic *E. coli*. The biogenesis of the P fimbriae of uropathogenic *E. coli* (UPEC) is dependent on *Ec*DsbA (Jacob-Dubuisson et al., 1994). Without functional P fimbriae, UPEC is unable to attach to urinary epithelial cells and is avirulent. P fimbriae consist of six subunit proteins that form a rod-like structure (Hultgren et al., 1991, 1993). The major P fimbrial rod subunit is PapA with PapG forming the adhesin while PapK, PapE, and PapF are the adaptor proteins. The PapC usher protein, which is integrated in the OM and acts as a scaffold protein, coordinates the polymerization of the fimbriae subunits. The periplasmic chaperone PapD binds to the individual subunits and targets them to the PapC usher protein in the OM (Hultgren et al., 1991, 1993). *Ec*DsbA is required for the correct folding of the chaperone protein PapD and the adhesion protein PapG (Jacob-Dubuisson et al., 1994). In addition, the formation of the Type IV pili or bundle forming pili (BFP) of Enteropathogenic *E. coli* (EPEC) is dependent on *Ec*DsbA. The BFP mediates adhesion and colonization of intestinal epithelial cells and a form of movement referred to as twitching motility (Pelicic, 2008). *Ec*DsbA is required for the formation of a single intramolecular disulfide bond in BfpA, the major subunit of BFP (Zhang and Donnenberg, 1996; Vogt et al., 2010). In the absence of *Ec*DsbA, BfpA is rapidly degraded in the periplasm, resulting in an inability of EPEC to form functional BFP (Zhang and Donnenberg, 1996). Lastly, flagella are complex cell surface organelles that mediate bacterial motility. In *E. coli*, *Ec*DsbA catalyzes the formation of a disulfide bond in FlgI, the flagellar P ring motor protein (Dailey and Berg, 1993). This critical disulfide is required for the biogenesis of functional flagella (Hizukuri et al., 2006). As a result, *Ec*DsbA is required for the cellular spread of pathogenic *E. coli* and attachment to host cells.

The Type III secretion system is required for secretion of effector proteins into host cells (Cornelis, 2006; Galan and Wolf-Watz, 2006). The Type III secretion system of Gram-negative bacteria is comprised of more than 20 proteins. It consists of a basal body anchored in the IM, linked to a needle that extends from the bacterial surface. The secretin, that is integrated in the OM forms a major structural component of the Type III secretion system and consists of 12–14 subunits arranged in an oligomeric ring-like structure (Bitter, 2003). In *E. coli*, *Ec*DsbA is required for the correct folding of the secretin and therefore the biogenesis of the Type III secretion system (Miki et al., 2008). An example of an effector protein in *E. coli* is the heat labile enterotoxin from enterotoxigenic *E. coli* (ETEC), that results in the acute diarrhoea associated with an ETEC infection. The heat labile toxin is an AB_5_ toxin that contains a critical disulfide bond in the B monomer that is required for oligomerization of the subunits (Yu et al., 1992; Wulfing and Rappuoli, 1997).

Recently, the oxidoreductases *Ec*DsbA and *Ec*DsbC have been shown to be essential for the biogenesis of *E. coli* LPS. Specifically, the highly conserved and essential OMP, LptD, contains a critical disulfide bond that is required for its role in integration of newly synthesized LPS in the OM of *E. coli* (Denoncin et al., 2010). LPS constitutes 70% of the outer leaflet of the OM and plays a significant role in the virulence of *E. coli*. The establishment of this link between periplasmic oxidoreductases and LPS biogenesis suggests a much larger role for oxidoreductases in OM formation in *E. coli*. The processes involved in LPS biogenesis and the role of LPS in virulence of *E. coli* have been extensively studied.

### Biosynthesis and transport of LPS to the OM in *E. coli* and role in pathogenesis

**Figure 3:**
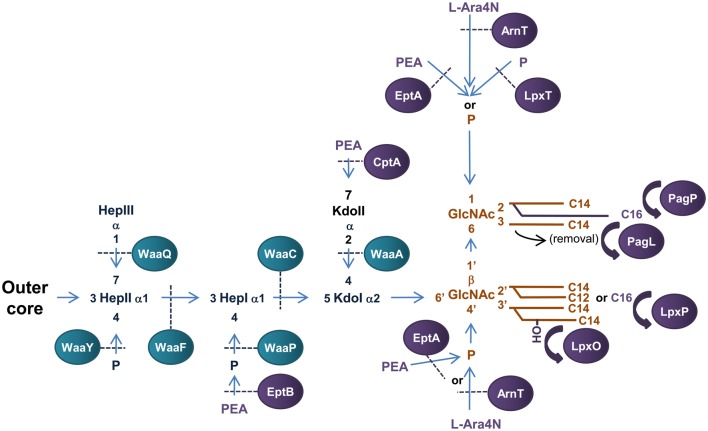
**Structure and biosynthesis of *E. coli* LPS.** The conserved Lipid A region is in orange while the conserved inner OS core region is in dark blue and variable modifications to these regions are in purple. Glycosyltransferases that form the inner OS core backbone are denoted by the blue boxes, and enzymes that modify the structure are in purple. The number of carbons present in each acyl chain is denoted by C followed by a number. Structure and biosynthesis of the lipid A region is reviewed in detail elsewhere (Raetz and Whitfield, 2002). Refer to text for details on enzyme functions.

The outer leaflet of the Gram-negative OM is composed mainly of LPS which forms a highly protective barrier to the external environment. The structure of the archetypal model of LPS of *E. coli* consists of three distinct regions; lipid A which forms the hydrophobic membrane anchor, core oligosaccharide (OS) and repeating polysaccharide or O-antigen (Raetz and Whitfield, 2002) (Figure 3). The lipid A is the toxic component of bacterial LPS (Galanos et al., 1985) while the O antigen provides a variable hydrophilic surface layer that can mask underlying antigenically-conserved core epitopes (Stenutz et al., 2006). The conserved OS core region of LPS consists of two L-*glycero*-D-*manno*-heptose (Hep) and two 3-deoxy-D-*manno*-octulosonic acid (Kdo) residues attached to lipid A. Various sugars can be added to the Hep_2_-Kdo_2_-lipid A structure to complete the formation of the inner OS core (lipid A proximal) and outer OS core (attachment site for O antigen) regions of LPS. These additions are the basis of core typing in *E. coli* while the composition of the O antigen attached to the outer core of LPS is the basis of antigenic variation.

#### Biosynthesis of E. coli LPS

Biosynthesis of *E. coli* LPS is initiated in the cytoplasm with the synthesis of Kdo_2_—lipid A region of LPS, which is conserved among Gram-negative bacteria and is the minimum structure required for growth in *E. coli* (Galloway and Raetz, 1990; Onishi et al., 1996). Construction of the Kdo_2_-lipid A region of LPS has been extensively studied in *E. coli* and is reviewed elsewhere (Raetz and Whitfield, 2002). This structure is then further modified to form the core OS—lipid A region before translocation across the IM upon which the O-antigen is added and the complete LPS transported to the OM.

The conserved inner OS core-lipid A structure of *E. coli* LPS consists of Hep_3_-Kdo_2_-lipid A with the HepI and HepII residues phosphorylated at the 4′ positions (Raetz and Whitfield, 2002). To construct the Hep_3_-Kdo_2_-lipid A structure, the α1,5 heptosyltransferase, WaaC, transfers a heptose residue to KdoI of Kdo_2_-lipid A to form Hep_1_-Kdo_2_-lipid A (Gronow et al., 2000; Zamyatina et al., 2000). The α1,3 heptosyltransferase, WaaF, then catalyzes the transfer of the second heptose residue to HepI to form the basic conserved LPS inner core structure of Hep_2_-Kdo_2_-lipid A (Gronow et al., 2000; Zamyatina et al., 2000). The LPS kinase, WaaP, then phosphorylates the 4′ position of HepI which is necessary for any further modification of the core structure (Yethon et al., 1998; Yethon and Whitfield, 2001). In fact, mutants in *waaP* exhibit a deep rough phenotype and are avirulent (Yethon et al., 2000a,b). Upon phosphorylation of HepI by WaaP, the LPS kinase, WaaY catalyzes the transfer of phosphate to HepII. This is followed by the addition of a heptose residue to HepII by the α1,7 heptosyltransferase, WaaQ (Yethon et al., 1998). This step completes the formation of the conserved inner OS core-lipid A of *E. coli* LPS.

There are five known core types (R1, R2, R3, R4, and K-12) based on the variable regions of the inner and outer OS core regions of *E. coli* LPS, the structure and biogenesis of which has been reviewed elsewhere (Raetz and Whitfield, 2002). An interesting example of a variable addition to the inner OS core region is the addition of a third Kdo residue to the 4′ position of KdoII of the inner OS core of *E. coli* core types R2 and K-12 catalyzed by the α-2,4 Kdo transferase, WaaZ (Frirdich et al., 2003). However, the core types are based mainly on the more variable outer core OS regions of *E. coli* LPS. The first addition to the outer core OS that is common to all core groups is the addition of glucose (Glc) to the 3′ position of HepII by the α1,3-glucosyltransferase, WaaG (Creeger and Rothfield, 1979; Heinrichs et al., 1998b). The *E. coli* LPS core type R1 has been extensively studied and the enzymes involved in the construction identified (Vinogradov et al., 1999; Raetz and Whitfield, 2002). The outer core OS region of the R4 core type differs from that of R1 by the presence of a β1,3 linked galactose (Gal) residue on GlcII instead of a β1,3 linked Glc from which the polysaccharide O antigen extends. These residues are transferred to the 3′ position of GlcII by the β1,3 galactoyltransferase, WaaX, and the β1,3 glucosyltransferase, WaaV, respectively (Heinrichs et al., 1998a). WaaX and WaaV are therefore important R4 and R1 core determinants. The site of polysaccharide O antigen attachment to the outer OS core also differs between core types. The ATP binding cassette (ABC) transporter MsbA mediates translocation of completed OS core-LPS across the IM to the site of O-antigen ligation in the periplasm (Karow and Georgopoulos, 1993; Zhou et al., 1998; Doerrler et al., 2001).

The polysaccharide O antigen forms the most structurally diverse region of *E. coli* LPS with 164 groups currently identified. The O antigen can exist as homopolymers or heteropolymers, they can be linear or branched, and can differ in anomeric configuration and linkage positions of the different sugar residues. Generally, the O antigen of *E. coli* LPS consists of 10–25 repeating subunits containing 2–7 sugar residues (Stenutz et al., 2006). Biosynthesis of O antigen begins in the cytoplasm with the synthesis of the O antigen subunits.

The O-antigen subunits are synthesized on the lipid carrier undecaprenyl diphosphate by glycosyltransferases using sugar nucleotides as donors (reviewed in Samuel and Reeves, 2003). Briefly, in *E. coli*, there are two synthesis models for polymerization and delivery of the O-antigen polymer to the site of ligation to OS core-LPS at the periplasmic surface of the IM. These are known as the Wzy-dependant and the ABC transporter dependant pathways, which vary in the machinery used, location of O-antigen polymerization and manner of translocation across the IM (Whitfield, 1995). In the Wzy-dependant pathway of O antigen synthesis, the O antigen subunits are flipped across the IM by the putative transporter Wzx (Liu et al., 1996; Feldman et al., 1999). Polymerization of the O-antigen subunits occurs on the periplasmic surface of the IM by the polymerase Wzy which transfers the nascent polymer to the reducing end of the new subunit (Robbins et al., 1967; Marolda et al., 2006) while Wzz determines the chain length modality (extent of polymerization) (Batchelor et al., 1991; Whitfield et al., 1997). In the ABC transporter dependant pathway, polymerization occurs at the cytoplasmic surface of the IM by sequential addition of glycosyl residues to the non-reducing terminus of the growing polymer (Robbins et al., 1967). The O antigen is then transported across the IM by the ABC transporter composed of Wzm and Wzt (Bronner et al., 1994; Kido et al., 1995). O antigen polymers synthesized by the Wzy-dependant pathway are more common and tend to be heteropolymers with branched repeating structures while O antigens produced by the ABC transporter pathway are generally simpler linear polymers (Stenutz et al., 2006). At the periplasmic side of the IM the rough LPS and polymerized O-antigen ligate to form LPS, a reaction catalyzed by the ligase, WaaL (Abeyrathne et al., 2005). The LPS is then ready for transport to the OM, during which certain modifications to the inner core and lipid A components may occur that impact on pathogenesis of the microorganism.

#### Transport of LPS to the OM: LPS transport (Lpt) pathway in E. coli

Unlike the LPS biogenesis pathway, transport of LPS through the periplasm to the OM is much less understood. Seven Lpt (LPS transport) proteins (A through G) have been implicated in transport of LPS across the periplasm to the OM. It is believed that LPS is extracted from the IM by the ABC transporter complex comprised of LptB, LptC, LptF, and LptG (Narita and Tokuda, 2009) and transferred to the periplasmic protein LptA (Sperandeo et al., 2007; Tran et al., 2008) which facilitates transfer across the periplasm to the OM assembly site composed of LptD and LptE (Wu et al., 2006; Chng et al., 2010b). The essential IM associated ABC transporter protein, LptB, has been implicated in extraction of LPS from the IM (Sperandeo et al., 2006, 2007). LptB (26.6 kDa) is predicted to contain the ATP binding fold but unlike other members of the ABC transporter superfamily is soluble and does not contain a transmembrane domain, suggesting that there are other proteins that interact with LptB to form the ABC transporter complex. In addition, LptB, was found in a ~140 kDa IM complex with interacting partners of unknown identity (Stenberg et al., 2005). Bioinformatic exploration identified LptF and LptG as the probable interacting partners (Ruiz et al., 2008) and LptF, LptG, and LptB were co-purified with the IM protein LptC (Narita and Tokuda, 2009) also implicated in LPS transport (Sperandeo et al., 2008). In this study, the purified LptB/C/F/G complex was also confirmed to have ATP hydrolytic activity *in vitro* (Narita and Tokuda, 2009). LptC has been shown to bind LPS *in vitro* suggesting that LptC contains a LPS binding motif (Tran et al., 2010). Once LPS is extracted from the IM it is proposed that LptC transfers LPS to the periplasmic protein LptA which facilitates transfer of LPS to the OM assembly site LptD/E (Sperandeo et al., 2007; Tran et al., 2008). LptA is an essential soluble periplasmic protein that has been implicated in LPS transport (Sperandeo et al., 2007, 2008). LptA interacts specifically with the lipid A region of LPS and relieves LptC of LPS *in vitro* (Tran et al., 2008, 2010) supporting the role in LPS transport from LptC in the LptB/C/F/G transporter in the IM to the OM assembly site LptD/E. The highly conserved and essential OMP LptD (formally known as Imp) is required for LPS transport to the cell surface (Braun and Silhavy, 2002; Bos et al., 2004) and interacts with the lipoprotein LptE forming an OM complex (Wu et al., 2006; Chng et al., 2010b). In *E. coli*, depletion of either LptD or LptE prevents newly synthesized LPS reaching the outer leaflet of the OM of *E. coli* (Wu et al., 2006) and LptE has been shown to interact specifically with LPS (Chng et al., 2010b). In *E. coli*, LptD is transported to the OM assembly site (the Bam complex, formerly the YaeT complex) by the periplasmic chaperone SurA where it is inserted into the OM (Wu et al., 2006; Denoncin et al., 2010).

There are two models proposed for the mechanism by which LptA facilitates the transfer of LPS from the IM ABC transporter LptB/C/F/G to the OM assembly site LptE/F. In one model, LptA acts as a molecular chaperone that forms a soluble complex with LPS to shuttle it from the IM to the OM. This model is analogous with the Lol-mediated lipoprotein transport system where the ABC transporter complex LolCDE facilitates the release of lipoproteins from the IM to the periplasmic LolA that transfers the lipoproteins to the OM assembly site LolB (Tokuda, 2009). The fact that LptA binds lipid A *in vitro* supports the role of LptA as a periplasmic chaperone (Tran et al., 2008). In the second model, oligomers of LptA form a proteinaceous bridge spanning the periplasm by which LPS is transported from the IM to the OM assembly site. This bridge may or may not be located at membrane adhesion patches known as Bayers patches. The fact that LptA forms filaments when crystallized in the presence of LPS supports this theory (Suits et al., 2008) as does the presence of a conserved OstA domain (Pfam database http://www.sanger.ac.uk//cgibin/Pfam/, accession no. PF03968) in LptA that is present in the periplasmic N-terminal of LptD (Sperandeo et al., 2006). In addition, it has been shown that LPS transport to the OM continues in spheroplasts when most of the periplasmic contents were lost (Tefsen et al., 2005). Most recently, all seven Lpt proteins have been co-purified in a membrane fraction containing both IM and OM fractions (Chng et al., 2010a). All of these data support the theory that the Lpt machinery forms a trans-envelope bridge to facilitate LPS transport from the IM to the OM.

#### Modifications of E. coli LPS and role in pathogenesis

Periplasmic modifications to *E. coli* LPS basic structure can play an important role in adaptation and pathogenesis of the microorganism (Trent et al., 2006; Raetz et al., 2007). At this stage, it is not known how these processes interact with the model for transport of LPS across the periplasm. Periplasmic alterations to *E. coli* LPS include changes to the acylation pattern and phosphate groups of lipid A. The phosphate groups attached to the disaccharide backbone of lipid A of *E. coli* LPS can be modified by addition of phosphate, phosphoethanolamine, (PEA) or 4*-amino*-4-*deoxy*-L-arabinose (L-Ara4N) (Zhou et al., 1999, 2000), which play a major role in susceptibility to Cationic Anti-Microbial Peptides (CAMPs) (Gunn et al., 1998; Zhou et al., 2001; Lee et al., 2004). CAMPs are important components of innate immunity as they are ubiquitous and have a broad spectrum of bacterial killing. They bind the OM of Gram-negative bacteria through electrostatic interactions with the negatively charged phosphate headgroups of lipid A, upon which they transit across the periplasm and intercalate into the IM, forming pores resulting in lysis (Zasloff, 2002). As a result, modification of the phosphate groups attached to the disaccharide back bone of LPS can significantly influence interaction with and susceptibility to CAMPs.

The addition of PEA and L-Ara4N to the phosphate headgroups of lipid A reduces the surface negative charge, increasing resistance to polymyxin B, a model CAMP (Gunn et al., 1998; Zhou et al., 2001; Lee et al., 2004). Transfer of PEA and L-Ara4N to *E. coli* lipid A headgroups is catalysed by the IM bound periplasmic enzymes, EptA (*E. coli* PEA transferase A) and ArnT (L-Ara4N transferase), respectively. EptA (formally known as PmrC in *Salmonella*) is a member of the alkaline phosphatase super-family that have conserved core structures and active site residues (Galperin and Jedrzejas, 2001; Trent and Raetz, 2002; Lee et al., 2004; Naessan et al., 2008). It preferentially transfers PEA to the 1-position of the disaccharide backbone of lipid A (Zhou et al., 2000; Lee et al., 2004). EptA can also catalyse the transfer of PEA to the 4′ position of lipid A (Zhou et al., 2000, 2001), however, this has recently been shown to only occur once decoration of the 1 - phosphate group with PEA has occurred (Herrera et al., 2010) and is enhanced in the absence of ArnT (Zhou et al., 2001). Interestingly, EptA has recently been found in a protein complex with the cell division protein ZipA suggesting a role for EptA in *E. coli* cell division (Stenberg et al., 2005). In addition, an EptA homolog in *Campylobacter jejuni* has recently been found to transfer PEA to the flagellar rod promoting assembly and subsequent motility (Cullen and Trent, 2010) suggesting a larger role for this family of proteins in the decoration of bacterial surfaces. ArnT (also known as PmrK in *Salmonella*) preferentially transfers L-Ara4N to the 4′—position of lipid A (Zhou et al., 2000, 2001; Trent et al., 2001c). Similar to EptA, ArnT has the ability to catalyse transfer of L-Ara4N to both phosphate headgroups (Zhou et al., 2000, 2001). It should also be noted that addition of L-Ara4N to the lipid A of *Salmonella* LPS is more prevalent than PEA addition (Zhou et al., 2001) and has greater influence over resistance to CAMPs (Tamayo et al., 2005). Transfer of an additional phosphate group to the 1-position of the disaccharide backbone of *E. coli* lipid A to form the 1-diphosphate structure (1-PP) is catalyzed by the undecaprenyl phosphotransferase, LpxT (Touzé et al., 2008). LpxT phosphorylation of the 1 - position of lipid A increases the negative charge of LPS and competitively inhibits transfer of PEA to the same position by EptA therefore increasing susceptibility to CAMPs (Herrera et al., 2010).

Modification of fatty acyl chain conformation of lipid A of *E. coli* LPS can greatly affect bacterial virulence. The OM palmitoyltransferase PagP transfers palmitate from phospholipids in the OM to the 2′ position of the proximal glucosamine residue resulting in a hepta-acylated structure (Bishop et al., 2000). Increased lipid A acylation resulting from increased PagP expression is an important mechanism for resistance to CAMPs (Guo et al., 1998) and attenuation of the inflammatory response mediated by the TLR4 signal transduction pathway resulting in immune evasion (Loppnow et al., 1986; Kawasaki et al., 2004a,b). The fatty acyl chain conformation of *E. coli* lipid A can be modified by the 3-O-deacylase, PagL, which removes the acyl chain from the 3′ position of the proximal glucosamine residue (Trent et al., 2001b). Deacylation and palmitoylation of *E. coli* lipid A by PagL and PagP, respectively, has been shown to decrease the potency of lipid A as an inducer of TLR4-medaited signaling (Kawasaki et al., 2004a,b). In *E. coli*, an acyltransferase, termed LpxP, is expressed at low temperatures (12°C) and adds palmitoleate to the 2′ position of the distal glucosamine residue of lipid A. This enzyme effectively replaces LpxL activity and results in the addition of an unsaturated fatty acyl chain to lipid A, presumably to aid in the adjustment of the fluidity of the OM during cold shock (Carty et al., 1999). Lastly, the lipid A acyl chain conformation of *E. coli* LPS can be altered by the addition of a 2-hydroxymyristoyl group in place of myristate at the 3′ position of the distal glucosamine residue by the acyl transferase, LpxO (Gibbons et al., 2000).

The inner core OS of *E. coli* LPS can be modified by addition of PEA to the phosphate group attached to HepI and/or the 7′ position of KdoII (Kanipes et al., 2001; Reynolds et al., 2005; Tamayo et al., 2005). The PEA transferase EptB (*E. coli* PEA transferase B) catalyzes the transfer of PEA to the phosphate group attached to HepI when grown in the presence of Ca^+2^ (Kanipes et al., 2001; Reynolds et al., 2005). In turn, addition of PEA to KdoII of LPS appears to play a role in tolerance to elevated levels of calcium presumably by decreasing membrane permeability to the cation (Reynolds et al., 2005). The OS inner core of *E. coli* LPS can be modified by the addition of PEA to the 7′ position of KdoII (Yethon et al., 1998). In *S. enterica* Serovar Typhimurium, the PEA transferase, CptA, is responsible for this addition (Tamayo et al., 2005). While PEA modification of KdoII of LPS increases in polymyxin B resistant strains of *S. enterica* Serovar Typhimurium (Helander et al., 1994), a *cptA* mutant was found to be only slightly more susceptible to polymyxin B (Tamayo et al., 2005). Therefore, the function of PEA addition to KdoII of *E. coli* and *S. enterica* Serovar Typhimurium LPS is yet to be determined.

### Regulation of LPS biogenesis and periplasmic protein folding in *E. coli*

**Figure 4:**
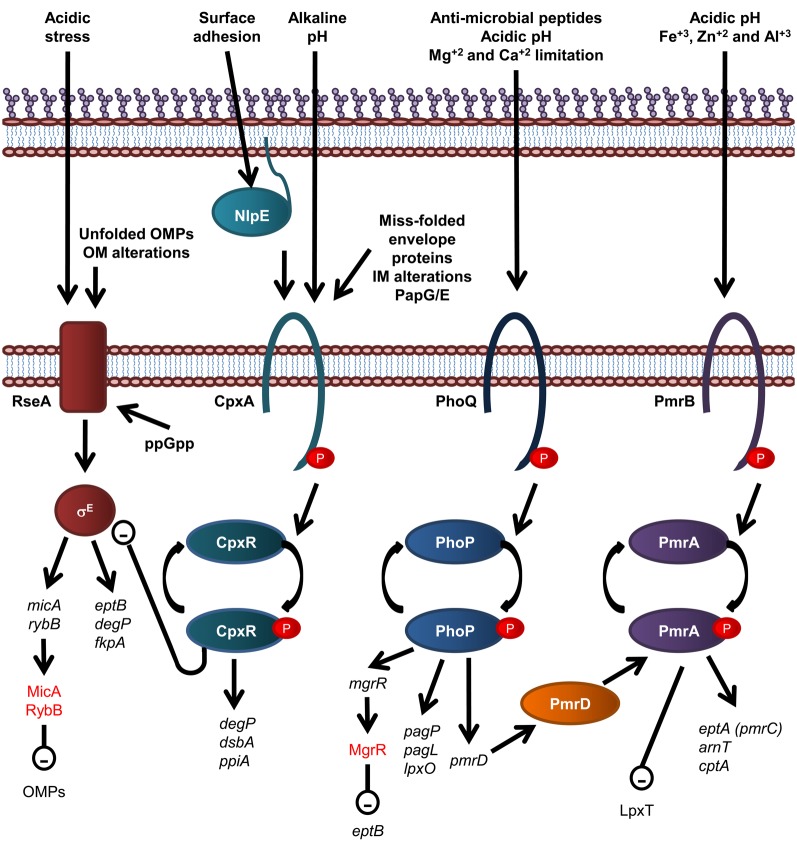
**Overview of regulatory systems involved in LPS biogenesis and periplasmic protein folding in *E. coli*.** The sensor histidine kinase and cognate response regulator protein of each of the CpxR/A, PhoP/Q, and PmrA/B two component systems are shown. The σE envelope stress response is also shown, where σE activation is inversely related to stability of the IM anti-sgma factor, RseA. Genes are in black text, lower case and italicized while proteins and sRNAs are in sentance case. sRNAs are in red text. Up-regulation of down stream processes is indicated by arrows while down-regulation is indicated by a minus sign within a circle.

There are a number of regulatory systems that act in concert to tightly regulate periplasmic protein folding and the biosynthesis of LPS in *E. coli* (Figure 4). The two component system is the most prevalent form of signal transduction enabling bacteria to alter cellular behavior in response to environmental cues (Stock et al., 2000). Typically, a two component system consists of a sensor histidine kinase that responds to specific signals by altering the phosphorylated state of a cognate response regulator protein. The vast majority of response regulators are DNA-binding transcription factors whose affinities for their target promoters are modulated by phosphorylation. Thus, by altering the phosphorylated state of a response regulator, a signal often modifies the gene expression profile of an organism (Beier and Gross, 2006; Mascher et al., 2006).

Modification of *E. coli* LPS is primarily under the control of the PmrA/B and PhoP/Q two component signal transduction systems, which respond to environmental cues that reflect that of the macrophage phagosome, such as low pH and the presence of antimicrobial peptides. The resulting modifications lead to enhanced survival in both host and non-host environments (Gunn et al., 1998). For example, in *S. enterica* serovar Typhimurium and *E. coli*, the substitution of lipid A headgroups with PEA and L-Ara4N, by EptA and ArnT, respectively, and the addition of PEA to KDOII of LPS by CptA, is under the control of the PmrA/PmrB two component histidine kinase/response regulator pair (Gunn et al., 1998; Zhou et al., 2001; Lee et al., 2004; Tamayo et al., 2005) which responds to mild acidic conditions and the presence of Fe^+3^, Zn^+2^, and Al^+3^ (Zhou et al., 1999, 2000; Trent et al., 2001a; Gibbons et al., 2005). These modifications lead to enhanced resistance to CAMPs (Helander et al., 1994; Gunn et al., 1998; Zhou et al., 2001; Lee et al., 2004). In addition, growth conditions that promote PmrA activation inhibit LpxT-dependent phosphorylation of lipid A, therefore promoting resistance to CAMPs, suggesting that PmrA/B regulates LpxT expression, although this effect does not involve the control of LpxT transcription (Herrera et al., 2010). The expression of PagP, PagL, and LpxO is under the control of the PhoP/Q two component histidine kinase/response regulator system in *S. Typhimurium* (Belden and Miller, 1994; Trent et al., 2001b; Kawasaki et al., 2004a) which is activated by the presence of CAMPs, low pH and Mg^+2^ and Ca^+2^ limitation (Garcia Vescovi et al., 1996; Soncini et al., 1996; Bader et al., 2003, 2005). To add another layer of complexity, the PhoP activated PmrD protein controls the PmrA/B two component system at a post-translational level as PmrD binds to phosphorylated PmrA, preventing dephosphorylation, leading to PmrA-mediated transcription (Kox et al., 2000; Kato and Groisman, 2004). Through this mechanism, the presence of CAMPs can be detected by the PhoQ sensory histidine kinase and lead to the PmrA-mediated transcription of genes; *eptA, arnT*, and *cptA*, as well as the PhoP-mediated transcription of *pagP*, which leads to LPS modification by EptA, ArnT, CptA, and PagP and subsequent increased resistance to CAMPs (Guo et al., 1997).

An alternative two component system that has been implicated in regulation of LPS modification is the SoxR/S superoxide and nitric oxide sensing two component system (Lee et al., 2009). The cognate response regulator, SoxS, binds the *waaY* promoter, leading to expression of the WaaY protein, which is responsible for phosphorylating the HepII of *E. coli* LPS inner core. WaaY expression is also regulated by the Multiple-Antibiotic Resistance marker, MarA, and is induced by salicylate, suggesting a role in antimicrobial resistance. In support of this, phosphorylation of the inner OS core of *E. coli* LPS has been implicated in conferring enhanced resistance against multiple drugs, possibly by increasing the integrity of the OM through cross-linking of neighboring LPS molecules via binding of divalent cations (Yethon et al., 1998; Yethon and Whitfield, 2001; Lee et al., 2009).

Recently, the PhoP/Q two component system has been shown to activate expression of the Mg^+2^-responsive RNA, MgrR, which down regulates expression of EptB (Moon and Gottesman, 2009), which provides an explanation as to why EptB-mediated PEA addition to *E. coli* LPS is increased in the presence of Ca^+2^, which has been shown to inhibit activation of the PhoP/Q two component system (Garcia Vescovi et al., 1996). Small non-coding RNAs (sRNAs), such as MgrR, are fast becoming recognized as key components of regulatory circuits that act as a fast acting “switches” to respond to stress signals (reviewed in Waters and Storz, 2009). The sRNAs, MicA and RybB, are activated in response to the σ^E^-envelope stress signaling to down regulate OMPs (Papenfort et al., 2006; Moon and Gottesman, 2009; Overgaard et al., 2009). Interestingly, the σ^E^-envelope stress response has been implicated in upregulation of EptB expression (Figueroa-Bossi et al., 2006), suggesting the possibility that MgrR participates in a negative feedback loop that controls EptB-mediated modification of LPS (Overgaard et al., 2009). The σE envelope stress signal system detects OM protein misfolding (Mecsas et al., 1993; Missiakas et al., 1996) and is activated by the well known cytoplasmic nutritional stress signal, guanosine 3′, 5′—bis—pyrophosphate (ppGpp) (Costanzo and Ades, 2006).

Periplasmic protein folding and degradation is controlled by the CpxR/A two component system (reviewed in Vogt and Raivio, 2012), which senses miss-folded envelope proteins, Pap pilus sub-unit over-expression, alkaline pH, and alterations of the IM (Nakayama and Watanabe, 1995; Jones et al., 1997; Mileykovskaya and Dowhan, 1997; Danese and Silhavy, 1998). CpxA is also activated by surface adhesion detected by the OM lipoprotein NlpE (Otto and Silhavy, 2002). The cognate response regulator, CpxR, induces the expression of periplasmic protein folding factors such as the oxidoreductase DsbA and the peptidyl-prolyl isomerize PpiA, and the periplasmic protease and chaperone DegP (Danese et al., 1995; Danese and Silhavy, 1997; Pogliano et al., 1997). Interestingly, CpxR negatively and directly regulates the expression of the *rpoErseABC* operon, which encodes the alternative sigma factor σ^E^, the mediator of an additional envelope stress response that detects OM protein misfolding (De Wulf et al., 2002; Price and Raivio, 2009).

## The Gram-negative cell envelope of *N. meningitidis*

*N. meningitidis* has a Gram-negative envelop which is structurally analogous to that of *E. coli*. However, there are some fundamental differences in the biology of these two pathogens. For example, unlike *E. coli* and other Gram-negative bacteria, LPS deficient strains of *N. meningitidis* are viable (Steeghs et al., 1998) which has led to significant advancements in the understanding of the machinery involved in LPS biosynthesis, transport, and assembly in the OM. Conversely, it has been recently shown that the protein isomerization pathway in *N. meningitidis* is essential for cell viability (Kumar et al., 2011), whilst this is not the case for *E. coli*. These observations suggest there are some important differences to be found in the genesis of the OM in *Neisseria sp*. Therefore, the remainder of this review will detail the differences in the oxidation/isomerization pathways and LOS biosynthesis pathway in *Neisseria sp*.

### The role of the periplasmic oxidoreductases in OM biogenesis and pathogenesis of *N. meningitidis*

**Figure 5:**
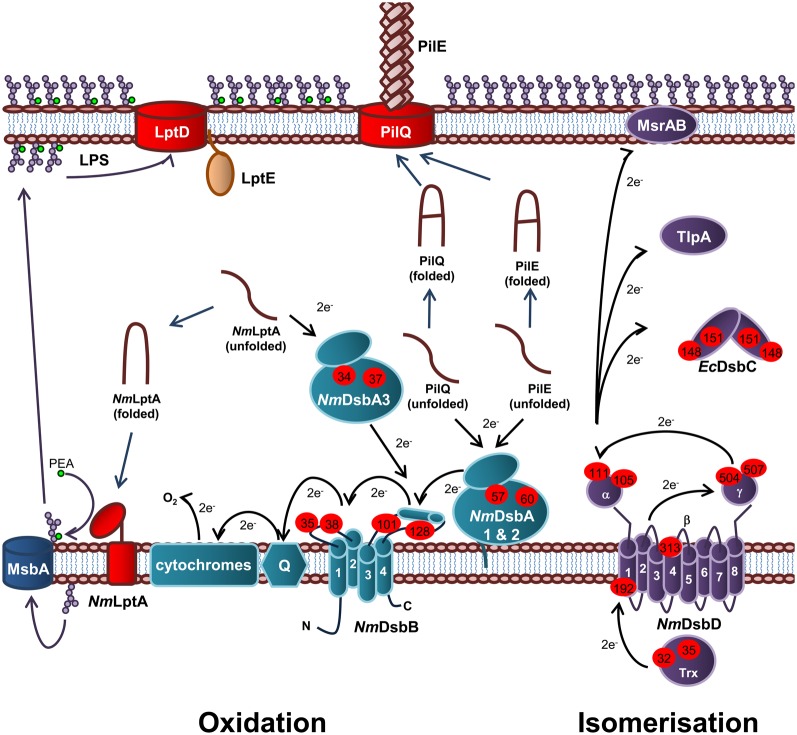
**Oxidation and isomerization pathways of periplasmic protein folding in *N. meningitidis* and contribution to virulence.** Oxidation pathway: The oxidoreductases *Nm*DsbA1 and *Nm*DsbA2 and IM bound lipoproteins that catalyze disulfide bond formation in the PilE and PilQ subunits of the Type IV secretion system. The soluble preiplasmic oxidoreductase *Nm*DsbA3 catalyzes disulfide bond formation in the periplasmic domain of the PEA transferase *Nm*LptA, which is responsible for PEA modification of LOS lipid A upon translocation across the IM by the transporter, MsbA, prior to transport across the periplasm and integration into the OM by LptD and LptE. *Nm*DsbA1, *Nm*DsbA2, and *Nm*DsbA3 are re-oxidized by the IM bound *Nm*DsbB. Under aerobic growth conditions, electrons flow from *Ec*DsbB to molecular oxygen via ubiquinone (Q) and cytochrome. Isomerization pathway: The isomerization pathway consists of a soluble periplasmic *Nm*DsbC, for which there is no known substrate and an IM bound *Nm*DsbD, which has also been implicated in the reduction of TlpA and MsrAB. *Nm*DsbD is reduced by thioredoxin in the cytoplasm. The oxidation pathway is in turquoise and the isomerization pathway is in purple. Cysteine residues are denoted by a red circle containing the residue number. The direction of electron transfer is shown by black arrows while protein folding reactions are shown by dark blue arrows and cellular processes are shown by dark purple arrows.

In *E. coli*, the oxidation pathway is a generalist one recognizing all chromosomally encoded proteins that require disulfide bonds and is supplemented with specialist protein oxidation pathways usually encoded on genetic islands or plasmids, while the isomerization pathway contains a generalist isomerase *Ec*DsbC and two substrate specific isomerases, *Ec*DsbG and *Ec*DsbE. In contrast, *N. meningitidis* appears to have adopted a specialist protein oxidation pathway which is partnered with a diversified protein isomerization pathway containing a wide variety of partners that interact with *Nm*DsbD. Specifically, *N. meningitidis* contains three DsbA homologs (*Nm*DsbA1, *Nm*DsbA2, and *Nm*DsbA3) as well as a single copy of *Nm*DsbB, *Nm*DsbC and *Nm*DsbD (Figure 5) (Sinha et al., 2004, 2008; Tinsley et al., 2004). These pathways have been shown to make important contributions to the virulence, pathogenesis, and viability of this microorganism.

#### Oxidation pathway of periplasmic protein folding in N. meningitidis

Each of the meningococcal DsbA enzymes have been shown to play a central role in virulence and the presence of at least one DsbA is required for normal growth of *N. meningitidis* (Tinsley et al., 2004; Sinha et al., 2008; Piek et al., 2012). The presence of *Nm*DsbA1 or *Nm*DsbA2 is required for formation of functional Type IV pilin and normal growth of *N. meningitidis* in the presence of reducing agents (Tinsley et al., 2004; Sinha et al., 2008). Type IV pili is a key virulence determinant of *N. meningitidis* as it initiates attachment to host cells and mediates DNA binding and uptake during natural transformation. Both *Nm*DsbA1 and *Nm*DsbA2 have been shown to introduce disulfide bonds into PilQ, the secretin which forms the pore through which the Type IV pili are extruded (Sinha et al., 2008). In addition, both oxidoreductases have been implicated in oxidation of the Type IV pili filament subunit, PilE (Tinsley et al., 2004). Recently, *Nm*DsbA3 has been shown to catalyze disulfide bond formation in the LPS PEA transferase, *Nm*LptA (Piek et al., 2012) and has therefore been implicated in resistance to CAMPs in *N. meningitidis*. Interestingly, *Nm*DsbA3 is unable to compensate for the loss of *Nm*DsbA1 and *Nm*DsbA2 (Tinsley et al., 2004; Sinha et al., 2008) and conversely, *Nm*DsbA1 and *Nm*DsbA2 are unable to compensate for the lack of *Nm*DsbA3 (Piek et al., 2012), suggesting that these enzymes are able to recognize and discriminate between substrates. The meningococcal DsbA enzymes were also found to preferentially interact with different *Ec*DsbA substrates when used to complement the *E. coliΔdsbA* mutant strain JCB571 (Sinha et al., 2004). While all three enzymes were able to restore resistance to the reducing agent dithiothreitol (DTT) only *Nm*DsbA1 restored motility to JCB571 and only *Nm*DsbA2 restored alkaline phosphatase activity (Sinha et al., 2004). However, *Nm*DsbA3 was able to oxidize FlgI and restore motility when over-expressed (Vivian et al., 2008). All three of the oxidoreductases have been shown to have oxidoreductase activity *in vitro* (Vivian et al., 2008, 2009; Lafaye et al., 2009).

Unlike *Ec*DsbA, *Nm*DsbA1 and *Nm*DsbA2 are membrane bound lipoproteins which share 78% amino acid identity with each other. In contrast, *Nm*DsbA3 is a soluble periplasmic oxidoreductase sharing 57% and 51% amino acid identity with *Nm*DsbA1 and *Nm*DsbA2, respectively (Sinha et al., 2004; Tinsley et al., 2004). All three of the meningococcal oxidoreductases share around ~20% amino acid identity with *Ec*DsbA (Sinha et al., 2004). Crystals of the soluble domain of *Nm*DsbA1 [PDB3A3T (Vivian et al., 2009) and PDB 3DVW (Lafaye et al., 2009)] and *Nm*DsbA3 [PDB 2ZNM (Vivian et al., 2008) and PDB 3DVX (Lafaye et al., 2009)] have been solved revealing a similar tertiary structure to *Ec*DsbA with a thioredoxin domain containing the thioredoxin-like fold and the active site motif CXXC with an inserted α-domain. Uniquely, *Nm*DsbA3 contains an active site motif of CVHC in an open loop conformation (Vivian et al., 2008) while *Nm*DsbA1 and *Nm*DsbA2 (modeled on *Nm*DsbA) contain the canonical active site of CPHC located at the N-terminus of the first α-helix of the thioredoxin domain, which is a similar in arrangement to *Ec*DsbA (Lafaye et al., 2009; Vivian et al., 2009). All three enzymes are extremely oxidizing with a standard redox potential of ~−80 mV (Vivian et al., 2008, 2009; Lafaye et al., 2009) compared to that of *Ec*DsbA (~−120 mV) (Huber-Wunderlich and Glockshuber, 1998). The Thr residue immediately preceding *cis*-Pro residue corresponding to position 151 in *Ec*DsbA, has been shown to confer this higher oxidizing power of these enzymes (Lafaye et al., 2009; Ren et al., 2009).

#### Isomerization pathway of periplasmic protein folding in N. meningitidis

Unlike *E. coli*, the isomerization pathway of *N. meningitidis* is essential (Kumar et al., 2011) suggesting a fundamental difference in this biochemical pathway and a key role in meningococcal lifestyle and virulence. *N. gonorrhoeae* contains a periplasmic thioredoxin-like protein, TlpA, which influences susceptibility to oxidative stress and the ability to survive in cervical epithelial cells (Achard et al., 2009). It has been suggested that TlpA is kept in a reduced state by DsbD (Achard et al., 2009). The substrate of TlpA is not known but is thought to be the methionine sulfoxide reductase (MsrAB), which repairs methioinine residues which have been attacked by reactive oxygen species (ROS). MsrAB is unusually located in the OM of *N. gonorrhoeae*, instead of in the cytoplasm as it is in other Gram-negative bacteria (Skaar et al., 2002). However, other work in this area has proposed that MsrAB is itself a direct redox partner of *Nm*DsbD (Brot et al., 2006). Therefore, in *Neisseria sp*. there has been a diversification of redox partners for *Nm*DsbD, all of which relate to the repair of proteins damaged due to oxidative stress.

### Biosynthesis and transport of LOS to the OM of *N. meningitidis*

**Figure 6:**
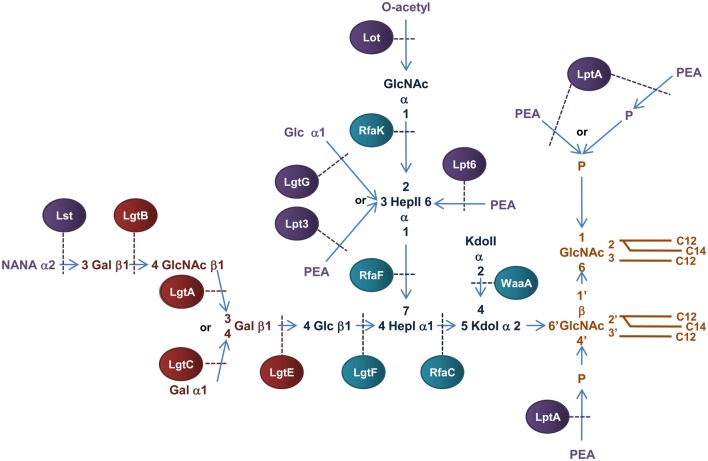
**Structure and biosynthesis of *N. meningitidis* LOS.** Conserved Lipid A region is in orange while the conserved inner OS core region is in dark blue and the variable α-chain in maroon. Variable modifications to these regions are in purple. Glycosyltransferases that form the inner OS core backbone and α-chain are denoted by the blue and light maroon boxes, respectively. Enzymes that modify the structure are in purple. The number of carbons present in each acyl chain is denoted by C followed by a number. Structure and biosynthesis of the lipid A region is reviewed in detail elsewhere (Raetz and Whitfield, 2002). Refer to text for details on enzyme functions.

In contrast to *E. coli* LPS, *N. meningitidis* does not produce an endotoxin with repeating O-antigen and is therefore more appropriately named lipooligosaccharide (LOS) (Jennings et al., 1980; Tsai et al., 1987). Meningococcal LOS consists of an inner OS core region attached to lipid A and a variable outer OS core region (α-chain) (Figure 6). The inner OS core consists of two Hep residues and two Kdo residues that can be decorated with variable sugar additions. Biosynthesis of meningococcal LOS is initiated in the cytoplasm with synthesis of the inner OS core attached to lipid A. The variable α-chain is then synthesized by sequential addition of sugars to HepI of the inner core and the completed LOS is then translocated to the periplasmic surface of the IM, transported across the periplasm and integrated into the OM.

#### Biosynthesis of meningococcal LOS

Biosynthesis of the Kdo_2_-lipid A region of *N. meningitidis* LOS is similar to the biogenesis of *E. coli* LPS except that one lauroyl residue is transferred to the 2′ position of each glucosamine unit resulting in a symmetrical molecule (Kulshin et al., 1991, 1992). Meningococcal LOS is synthesized by sequential transfer of sugars to the Kdo_2_-lipid A structure at the cytoplasmic surface of the IM. There are many enzymes known to facilitate this process.

The basic OS core region of meningococcal LOS consists of Glcβ1,4 (GlcNAc_1_)-Hep_2_-Kdo_2_ attached to lipid A (Kahler and Stephens, 1998). Transfer of a ADP-Hep to KdoI of the conserved Kdo_2_-lipid A structure, mediated by the α1,5 heptosyltransferase, RfaC, forms Hep_1_-Kdo_2_-lipid A. The α1,3 heptosyltransferase, RfaF, then catalyzes the transfer of the second ADP-Hep to HepI to form Hep_2_-Kdo_2_-lipid A (Kahler and Stephens, 1998) which forms the minimum LOS structure required for invasion of nasopharyngeal epithelial cell lines (Plant et al., 2006). The structure of meningococcal LOS then diverges from the *E. coli* model by addition of *N*-Acetylglucosamine (GlcNAc) to the 2′ position of HepII and Glc to the 4′ position of HepI (Kahler et al., 1996b). The transfer of UDP-GlcNAc to position 2′ of HepII is catalyzed by the α1,2 *N*-acetylglucosamine transferase, RfaK. This step results in the formation of GlcNAc_1_-Hep_2_-Kdo_2_-lipid A and is a necessary prerequisite for further LOS biosynthesis (Kahler et al., 1996a). The transfer of UDP-Glc to the 4′ position of HepI is mediated by the β1,4 glycosyltransferase, LgtF, resulting in the conserved core region of meningococcal LOS, Glc β1,4 (GlcNAc_1_)-Hep_2_-Kdo_2_-lipid A (Kahler et al., 1996b). From the terminal Glc of this structure the variable α-chain can extend.

Meningococcial LOS can express a long α-chain composed of lacto-*N*-neotetraose (Gal β1,4→GlcNAcβ1,3→Galβ1,4→ or LNT) or a short α-chain composed of either Gal α1,4→Galβ1,4→ or Galβ1,4→ and is dependent on expression of genes in the lipooligosaccharide glycosyltransferase (*lgtABCE*) locus (Wakarchuk et al., 1996). Specifically, switching between the α-chains is achieved by high frequency mutation within the poly-G tract of *lgtA* and *lgtC*, a process referred to as phase variation (Jennings et al., 1999; Zhu et al., 2002). First, the β1,4 galactosyltransferase, LgtE, catalyzes transfer of UDP-Galactose to the terminal Glc attached to HepI. LNT formation then depends on the phase variable expression of the β1,3 *N*-acetylglucosamine transferase, LgtA, that transfers UDP-GlcNAc to the 3′ position of the terminal Gal of Galβ1,4→Glcβ1,4→HepI of LOS. The β1,4 galactosyltransferase, LgtB, then transfers UDP-Galactose to the 4′ position of the GlcNAc resulting in the LNT structure (Jennings et al., 1995; Wakarchuk et al., 1996). In the absence of *lgtA* expression, the α-chain can terminate at Galβ1,4→ or depending on the phase variable expression of *lgtC*, the α1,4 galactosyltransferase LgtC can transfer UDP-Galactose to the 4′ position of the terminal Gal residue resulting in the formation of the short α-chain, Galα1,4→Galβ1,4→ (Wakarchuk et al., 1998).

Variable additions to the LOS inner OS core can occur prior to transport across the IM. The terminal GlcNAc attached to the 2′ position of HepII can be O-acetylated by the phase variable lipooligosaccharide O-acetyltransferase, Lot, forming the γ-chain extension (Kahler et al., 2006) and the O-3 position of HepII can be modified by transfer of a Glc residue mediated by the α1,3 glycosyltransferase, LgtG (Banerjee et al., 1998). The *lgtG* gene is phase variable (Jennings et al., 1999) but is part of a small genetic island that is absent in some isolates (Jennings et al., 1999).

#### Transport of LOS to the OM: Lpt pathway in N. meningitidis

Meningococcal LOS is transported across the IM by the IM ABC transporter MsbA (Karow and Georgopoulos, 1993; Zhou et al., 1998; Doerrler et al., 2001) to the periplasm and is targeted to the OM by Lpt proteins similar to that in *E. coli*. One notable difference is that in *N. meningitidis*, LptE is not essential to LOS transport and has been suggested to have a chaperone-like role in LptD biogenesis (Bos and Tommassen, 2011). Interestingly, unlike *E. coli* where the periplasmic chaperone SurA is required for transport of LptD to the OM (Vertommen et al., 2009; Denoncin et al., 2010), SurA appears not to play a role in OM biogenesis of *N. meningitidis* (Volokhina et al., 2011). Upon integration into the OM, meningococcal LOS can be modified by the transfer of *N*-acetylneuraminic acid (NANA) to the 3′ position of the terminal Gal of LNT by the LOS α2,3 sialyltransferase, Lst, resulting in an α-chain extension of NANAα1,3→Galβ1,4→GlcNAc→β1,3→Galβ1,4→ (Mandrell et al., 1991; Gilbert et al., 1996).

#### Periplasmic modifications of meningococcal LOS

The lipid A and inner core regions of meningococcal LOS can be modified by the addition of PEA during transit through the periplasm to the OM. The OS core of meningococcal LOS can be modified by addition of PEA to the 3′ or 6′ positions of HepII catalysed by the enzymes, Lpt3 (LOS PEA transferase 3) and Lpt6 (LOS PEA transferase 6), respectively (Mackinnon et al., 2002; Wright et al., 2004; Kahler et al., 2005). Transfer of PEA to the 3′ carbon of HepII by Lpt3 can be competitively inhibited by the addition of α1–3 Glc to the same position by LgtG in the cytoplasm before transport into the periplasm (Banerjee et al., 1998; Mackinnon et al., 2002). In addition, acetylation of the terminal GlcNAc attached to the 2′ position of HepII in conjunction with an α-chain of LNT can inhibit PEA addition to the 3′ position of HepII (Kahler et al., 2006). Unlike *E. coli* and *Salmonella*, the lipid A phosphate headgroups of meningococcal LOS are not decorated with arabinose but exist as a mixture of “phosphoforms.” The basal diphosphorylated species of meningococcal LOS can be modified by the addition of a single PEA to the 4′ headgroup or by addition of PEA to both ends of the disaccharide backbone (never at the 1 position alone). The lipid A can also contain an additional phosphate residue at the 1 headgroup with or without PEA (Kulshin et al., 1992; Cox et al., 2003). In *N. meningitidis*, the PEA transferase *Nm*LptA catalyzes the transfer of PEA to the lipid A of LOS (Cox et al., 2003). While inactivation of *NmlptA* results in a total loss of PEA substitutions to lipid A, it is not known whether *Nm*LptA transfers one or both of the PEA groups to the lipid A headgroups (Cox et al., 2003).

#### Role of LOS in pathogenesis of N. meningitidis

The length and composition of the LOS α-chain is a major virulence determinant of *N. meningitidis*. Invasive isolates of *N. meningitidis* express an α-chain of LNT that mimics human paragloboside, increasing resistance to serum bactericidal activity (Moran et al., 1996; Harvey et al., 2001). An α-chain of LNT is therefore required for survival in the blood stream and the establishment of bacteraemia (Mackinnon et al., 1993; Moran et al., 1994; Virji et al., 1995). Sialylation of the α-chain further increases resistance to serum bactericidal activity (Kahler et al., 1998). In contrast, a short α-chain consisting of Galα1,4→Galβ1,4→ or β1,4 Gal→ allows colonization and invasion of the nasopharynx by invasive isolates (Moran et al., 1994). The ability to switch between carriage and invasive modes by the phase variable expression of α-chain biosynthesis genes *lgtA* and *lgtC* is therefore a major virulence determinant of *N. meningitidis*.

Periplasmic modifications of the lipid A and OS core of meningococcal LOS by PEA can mediate resistance to defensins at the site of colonization and attachment to host cells as well as influence survival in the blood stream and the potency of lipid A as an inducer of the host inflammatory immune response (Kahler et al., 1998; Ram et al., 2003; Tzeng et al., 2005; Takahashi et al., 2008). *Nm*LptA catalyzed addition of PEA to lipid A of meningococcal LOS is essential for resistance to CAMPs (Tzeng et al., 2005) and the ability to attach to nasopharyngeal epithelial cell lines (Takahashi et al., 2008). *Nm*LptA mediated addition of PEA groups to lipid A of LOS of the closely related pathogen *N. gonorrhoeae* results in increased resistance to complement mediated killing by normal human serum (Lewis et al., 2009), suggesting a possible role of PEA addition to lipid A in meningococcal serum resistance. In addition, the level of PEA decoration of lipid A can influence the potency of lipid A as an inducer of the host inflammatory immune response (Liu et al., 2010). Variable sugar additions to the carbons of HepII of the inner OS core of meningococcal LOS can influence resistance to complement mediated lysis and therefore play an integral role in meningococcal pathogenesis. The PEA groups attached to HepII of meningococcal LOS are a target for complement component C4b resulting in enhanced killing by the classical pathway of complement. Furthermore, the O-6 linked PEA groups are more efficient at binding the complement component, C4b, than the O-3 linked PEA (Ram et al., 2003). As a result modification of the inner OS core of meningococcal LOS, particularly decoration with PEA, is central to pathogenesis of *N. meningitidis*.

### Regulation of LOS biogenesis and periplasmic protein folding in *N. meningitidis*

**Figure 7:**
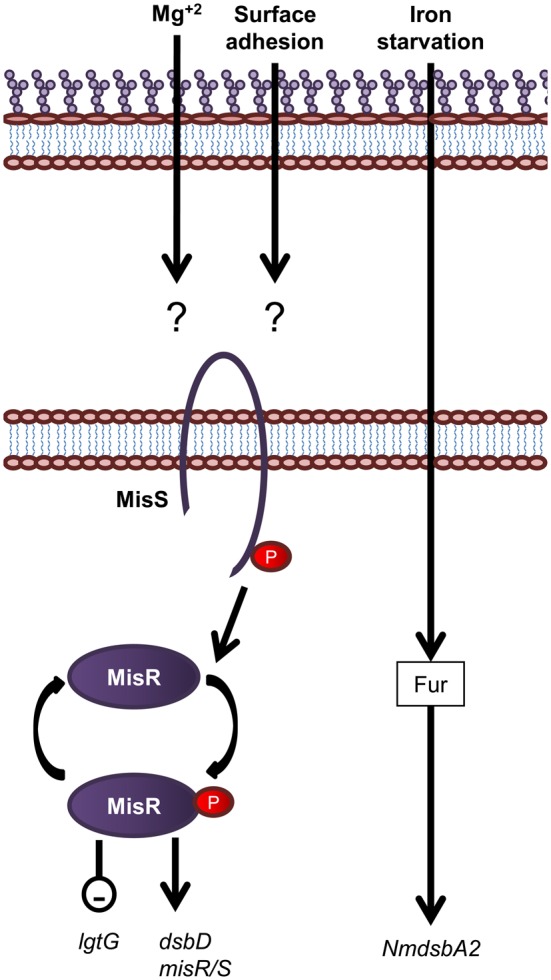
**Overview of regulatory systems involved in LPS biogenesis and periplasmic protein folding in *N. meningitidis*.** The sensor histidine kinase and cognate response regulator protein of the MisS/R two component system is shown. Expression of the oxidoreductase gene *NmdsbA2* is regulated by an unknown mechanism in response to iron. Genes are in black text, lower case and italicized while proteins are in sentence case. Up-regulation of downstream processes is indicated by arrows while down-regulation is indicated by a minus sign within a circle.

In contrast to *E. coli*, which encodes more than 30 two component systems (Oshima et al., 2002; Yamamoto et al., 2005), *N. meningitidis* contains only four predicted two component systems which is a feature common to obligate host pathogens from restricted environments (Parkhill et al., 2000; Tettelin et al., 2000). The two component system, MisR/MisS (meningococcal inner core structure), has been shown to be involved in LOS modification and periplasmic protein folding (Figure 7). The MisR/MisS histidine kinase/response regulator pair has been proposed to be analogous to the PhoP/PhoQ system of *E. coli* (Johnson et al., 2001; Newcombe et al., 2005). However, evidence for responses to different environmental cues suggests a different role. Inactivation of the MisR/MisS two component system in *N. meningitidis* increases sensitivity to CAMPs (Johnson et al., 2001; Tzeng et al., 2004), reduces haem utilization (Zhao et al., 2010) and causes attenuation in a mouse model of meningococcal infection (Newcombe et al., 2004; Rustam et al., 2006). While initial studies using *N. meningitidis* serogroup C strains, M96255789 and L91543, found the MisR/MisS two component system to be activated by Mg^+2^ limitation (Johnson et al., 2001; Newcombe et al., 2005), this has not been confirmed by more recent studies in *N. meningitidis* serogroup B strain, NMB (Tzeng et al., 2006). In addition, a separate study has shown that MisR/MisS is regulated in response to host cell contact (Jamet et al., 2009). The MisR/MisS two component system influences decoration of the inner OS core of meningococcal LOS. Inactivation of MisR resulted in increased expression of the LOS glycosyltransferase, LgtG, responsible for transferring a Glc residue to the 0–3 position of HepII (Tzeng et al., 2004). In addition, while there was no change in expression of the inner core PEA transferase enzymes, Lpt3 and Lpt6, the MisR mutant was completely devoid of PEA modifications of the inner core, which in turn resulted in increased sensitivity to CAMPs (Tzeng et al., 2004). In addition, transcription of *dsbD*, but not *dsbC* or other *dsb* genes, is positively regulated by the MisR/MisS (Tzeng et al., 2008; Kumar et al., 2011). Apart from the MisR/MisS circuit, the only other known regulatory circuit for the protein oxidation pathway is the *fur* (ferric uptake regulator) dependent regulation of *NmdsbA2* (Klee et al., 2000), which is upregulated in conditions of iron starvation (Grifantini et al., 2003).

Extensive transcriptome analysis of gene expression in *N. meningitidis* has not revealed any other regulatory circuits controlling the protein oxidation and isomerization or LOS biosynthesis pathways in response to specific conditions such as heat shock (Guckenberger et al., 2002), contact with eukaryotic cells (Grifantini et al., 2002a,b; Dietrich et al., 2003), exposure to human serum (Dietrich et al., 2003; Kurz et al., 2003), biofilm formation (Phillips et al., 2012), oxidative stress (Seib et al., 2007), or iron starvation (Grifantini et al., 2003; Basler et al., 2006). In addition, genes involved in LPS biogenesis and periplasmic protein folding are not regulated in response to the alternative sigma factors, σ^E^ (Gunesekere et al., 2006b; Huis in ‘T Veld et al., 2011) and σ^32^ (Du and Arvidson, 2006; Gunesekere et al., 2006a; Folster et al., 2009) of *N. meningitidis*. Lastly, the other functional two component systems of *N. meningitidis*, NarQ/NarP (NMB1249/NMB1250) and NtrY/NtrX (NMB0114/NMB0115), have not been found to be involved in the regulation of LPS biosynthesis or oxidative protein folding genes. Microarray analysis of the regulon of the two component system, NarQ/NarP, indicates that it regulates genes required for survival in anaerobic conditions in *N. gonorrhoeae* by activating the nitrite reductase, AniA (Lissenden et al., 2000; Overton et al., 2006; Whitehead and Cole, 2006). Although it is known that the NtrY/NtrX two component system, which is involved in nitrogen fixation and metabolism in *E. coli* (Pawlowski et al., 1991), is down-regulated in response to human whole blood in *N. meningitidis* (Echenique-Rivera et al., 2011), no transcriptome has been reported.

## The effect of genome reduction on OM biogenesis

Overall, the OM biogenesis pathways of LPS biosynthesis and protein oxidation and isomerization remain conserved in function in both Gram-negative species. However, in the commensal *E. coli*, the LPS pathway is essential to bacterial cell viability due to the loss of OM integrity (Galloway and Raetz, 1990; Onishi et al., 1996), whilst in *N. meningitidis*, the analogous pathway is not essential (Steeghs et al., 1998). Conversely, the protein oxidation and isomerization pathways which are required for the correct folding of virulence proteins and the protection of proteins in the periplasm from oxidative damage, are not essential in *E. coli*, but are essential for meningococcal viability (Kumar et al., 2011). Of great interest is the fact that both the endotoxin and protein oxidation/isomerization pathways are co-regulated in such a way as to maintain OM integrity. In *E. coli*, the number of environmental signals and hence regulatory networks involved in regulating these pathways are very diverse, whereas in the specialized pathogen *Neisseria sp*., only the regulatory network of the MisR/MisS two component system has been identified so far in performing this dual role. Unlike *E. coli* MsrABs and thioredoxins which are confined to the cytoplasm, neisserial MsrAB and TlpA are secreted to the outer membrane and periplasm, respectively, are both required for protection from oxidative stress and are regenerated by the protein isomerization pathway (Boschi-Muller et al., 2008). Since *Neisseria sp*. encounter high levels of ROS and hydrogen peroxide in mucosal secretions during colonization of these surfaces (Criss and Seifert, 2012), the expansion of redox partners for the protein isomerization pathway in this species may suggest that this aspect of the pathway has evolved through niche adaption. Future work to understand the role of redox partners of the protein isomerization pathways in niche adapted pathogens from other genera may uncover other interesting features.

### Conflict of interest statement

The authors declare that the research was conducted in the absence of any commercial or financial relationships that could be construed as a potential conflict of interest.
